# The development of anti-bullying, discrimination and harassment guidance: a survey among the Association of Surgeons in Training (ASiT) council members

**DOI:** 10.1308/rcsann.2023.0071

**Published:** 2023-11-06

**Authors:** M El Boghdady

**Affiliations:** Association of Surgeons in Training, UK

**Keywords:** Bullying, Discrimination, Harassment, Surgical trainees, Misconduct, Racial disparities

## Abstract

**Introduction:**

Surgical trainees have a reasonable expectation to feel safe and valued in their workplace. Previous reports proved that poor behaviour and misconduct existed in national health systems. This study aimed to conduct a survey among the Association of Surgeons in Training (ASiT) council members to identify the need for guidance to report bullying, discrimination and harassment for trainees who experienced any type of poor behaviour in the workplace.

**Methods:**

Data among executive and council members were collected. Questions were related to trainee demographics, level of training, specialties, and experience of, witnessed or reported poor behaviours including bullying, discrimination and harassment. We asked if participants lacked direction when experiencing poor behaviours, and if support strategies were needed such as a standardised guidance for reports.

**Results:**

A total of 58 survey responses were received: 55.17% of participants experienced bullying, 77.58% witnessed it and 67.25% did not report the incidents. Furthermore, 37.93% experienced discrimination, 62.07% witnessed it and 68.97% did not report. A total of 24.14% experienced sexual harassment, 29.69% witnessed it, while 72.41% did not report. Over 80% mentioned they need more guidance to support trainees. Almost all participants (98%) agreed that surgical trainees should be made aware of routes for reporting, and 88% agreed that ASiT should develop the guidance to support trainees against poor behaviours.

**Conclusion:**

Most of the trainees who experienced or witnessed poor behaviours did not report the incidents. A new standardised anti-bullying, anti-discrimination and anti-harassment guidance was developed based on our study results. We envisage that its use may play a role in eliminating misconduct in surgical training.

## Introduction

Surgical trainees have a reasonable expectation to feel safe and valued in their workplace. In a previous survey among National Health Service (NHS) staff, 421 (38%) reported experiencing one or more types of bullying, and 42% had witnessed others being bullied.^1^ Bullying behaviour has been defined as offensive, intimidating or insulting behaviour, an abuse or misuse of power through means that undermine or humiliate someone. Bullying was prevalent in the surgical domain among 1,412 surveyed surgical trainees.^2^ Bullying and witnessing bullying were associated with lower levels of psychological health and impaired job satisfaction, as well as with higher levels of intention to leave work.^3^

Discrimination has been defined as an unfair and unequal treatment of someone based on their race, ethnicity, gender, sexuality, disability or religious beliefs. The report Racism in Medicine surveyed UK doctors and medical students on their experience of racism in the medical profession and the workplace.^4^ The survey showed that widespread racism existed on a personal and institutional level. A total of 1,047 doctors (52%) reported experience of bullying in their workplace related to their ethnicity, and this was most often (68%) done by senior colleagues. Sixty per cent of 896 respondents mentioned that the racism they had experienced had negatively affected their wellbeing. NHS staff, particularly those working in London trusts, were exposed to unique levels of discrimination and harassment from their colleagues.^5^

A BMJ report defined forms of sexual harassment at workplace, which is an unwanted conduct of a sexual nature, which has the purpose or effect of violating someone’s dignity or creating an intimidating, hostile, humiliating or offensive environment for that individual. It mentioned that in hierarchal work structures like the NHS, it is often endemic.^6^ A UNISON study found that 8% of the surveyed NHS staff identified having been sexually harassed in the past 12 months, with behaviour ranging from rude sexual comments to rape.^7^ The British Medical Association (BMA) recorded that sexual harassment was most often committed by colleagues (54%), as opposed to other workers (24%) or patients (42%). Furthermore, women were disproportionately affected (81% of cases).^8^

A previous study discussed that the first step to work against any misconduct is to encourage victims to speak up and to seek guidance, when escalation takes place, and a formal report will be needed.^9^ However, in the previous NHS survey, two-thirds of the victims of such poor behaviours had tried to act when the bullying occurred, but most of them were dissatisfied with the outcomes.^1^ The main barriers to reporting were the perception that nothing would change and not wanting to be considered as troublemakers.^3^

The Association of Surgeons in Training (ASiT) is an educational charity promoting excellence in surgical training across all surgical specialties in the UK and Ireland. ASiT released a previous position statement highlighting the excess rates of undermining and bullying among surgical trainees, followed by a review of its recommendations.^10,11^ ASiT created the new council role in 2020 of equality, diversity and inclusion (EDI) officer. One of the roles of the equality and diversity officer is to raise awareness in surgical training about underrepresented groups and to promote excellence in surgical training for the benefit of patients and trainees irrespective of their race, gender, sexual orientation, disability and religion.

We aimed to study any experienced or witnessed poor behaviours among ASiT council members, identify the need for support strategies and to develop anti-bullying, anti-discrimination and anti-harassment (anti-BDH) guidance for trainees who experienced any type of misconduct or poor behaviour in the workplace.

## Methods

We collected questions relating to trainee demographics, level of training, specialties, and experience of, witnessing or reporting of any type of poor behaviours including bullying, discrimination or harassment. We asked if participants lacked guidance for those who experienced poor behaviours, and asked about support strategies and the possible need for a standardised guidance for reporting misconducts and poor behaviours, in addition to questions about proposed steps for escalation or resolving any raised concern of misconduct.

The questions were developed by the equality and diversity officer and piloted among ASiT executive members. The survey was then distributed via Surveymonkey® among all ASiT executive and council members. A reminder was issued after two weeks followed by weekly reminders in a WhatsApp group for council members. The survey was open for the entire month of January 2023.

No ethical concerns were identified during conception and execution of the study, and according to the Medical Research Council checklist, no specific ethical approval was required. Participation was voluntary, and completion of the online survey was accepted as informed consent. Responses were anonymously collected and imported into Excel (Microsoft Excel 2019) for analysis. Descriptive statistics and proportional data are provided using percentages. No comparative statistical analyses were required. This study has been reported in line with Standards for Reporting Qualitative Research (SRQR).^12^

## Results

A total of 58 survey responses were received (including 100% response rate from executive members and 80% from council members, respectively). All training regions and surgical specialties were represented.

*Demographics, level of training and specialities:*

Altogether, 52% were males and 48% females. We also included other gender options comprising transgender male, transgender female, non-binary, prefer not to say and prefer to self-describe.

Participants varied from medical students to post-certificate for completion of training (CCT): 22.4% were ST3, followed by 17% CT1 and 17% ST5.

Participants represented different specialties including general surgery (50% of respondents); other specialties included plastic surgery, cardiothoracic surgery, trauma and orthopaedic surgery, vascular surgery, paediatric surgery, urology and others (not specified).

*Questions on bullying-type behaviour:*

Have you experienced bullying-type behaviour?

**Fifty-five per cent** experienced bullying.

Have you witnessed bullying-type behaviour?

**Almost 78% witnessed** bullying.

Have you reported bullying-type behaviour?

**Sixty-seven per cent** did **not** report.

*Questions on discrimination-type behaviour:*

Have you experienced discrimination-type behaviour?

**Thirty-eight per cent** experienced discrimination.

Have you witnessed discrimination-type behaviour?

**Sixty-two per cent** witnessed discrimination.

Have you reported discrimination-type behaviour?

**Sixty-nine per cent** did **not** report.

*Questions on harassment-type behaviour:*

Have you experienced sexual harassment-type behaviour?

**Twenty-four per cent** experienced sexual harassment.

Have you witnessed sexual harassment-type behaviour?

**Almost 30%** witnessed sexual harassment.

Have you reported sexual harassment-type behaviour?

**Seventy-two per cent did not** report.

Questions on support strategies and development of new pathway are presented in Table 1.

**Table 1:** Results of the survey on support strategies and the development of the standardised guidance to report poor behaviours

Statements	Strongly agree (%)	Agree (%)	Neutral (%)	Disagree (%)	Strongly disagree (%)
Support strategies					
I believe there's lack of guidance for trainees to report poor behaviours, e.g. bullying, discrimination and harassment	25.86	48.28	18.97	6.90	0
I know how to guide trainees in my region if they face poor behaviours during their surgical training	3.45	31.03	36.03	20.69	8.62
I need more guidance to give support to trainees who experienced poor behaviours	25.86	55.17	12.07	5.17	1.72
Surgical trainees should be made aware of routes for reporting experiences of poor behaviours	56.90	41.38	1.72	0	0
Anti-bullying, discrimination and harassment strategies should be developed with key stakeholders	55.17	41.38	1.72	0	1.72
ASiT should develop a standardised pathway to support trainees against poor behaviours	41.38	46.55	12.07	0	0
Developing a new standardised anti-bullying, discrimination and harassment guidance					
I find the trainees should start by trying to informally resolve the incident with the offender	1.72	20.69	39.66	29.31	8.62
Trainees should escalate to educational supervisor, clinical supervisor, speak up guardian, clinical/medical director	32.76	58.62	8.62	0	0
I find the deanery should be aware of any reported poor behaviours in the regions	55.17	39.66	5.17	0	0
EDI officer should be involved in guiding reports/escalation of poor behaviours	13.79	51.72	27.59	5.17	1.72

We also asked the council members ‘I find further reports are needed to GMC in case of any unresolved incidents of poor behaviours’, and there was a consensus without any objection.

At the end of the survey, we asked the participants if there were any comments to add. Some of the comments were:

‘*I’ve been bullied and discriminated, witnessed sexual harassment and was not sure how to escalate. Trainees will need more guidance*’.

‘*I think it is extraordinary difficult to escalate poor behaviours. In my deanery it is rare and basically involves a handful of known individuals. Everyone knows how they are. I think it is wrong and unprofessional, but I feel nothing will be done till these individuals will retire*’.

‘*I’ve recently experienced bullying from consultant level, difficulties with his peers being close friends and TPD being close colleagues is a challenge for escalation. I think having ASiT council member to approach for escalation through different channels would be helpful*’.

**Figure 1:**
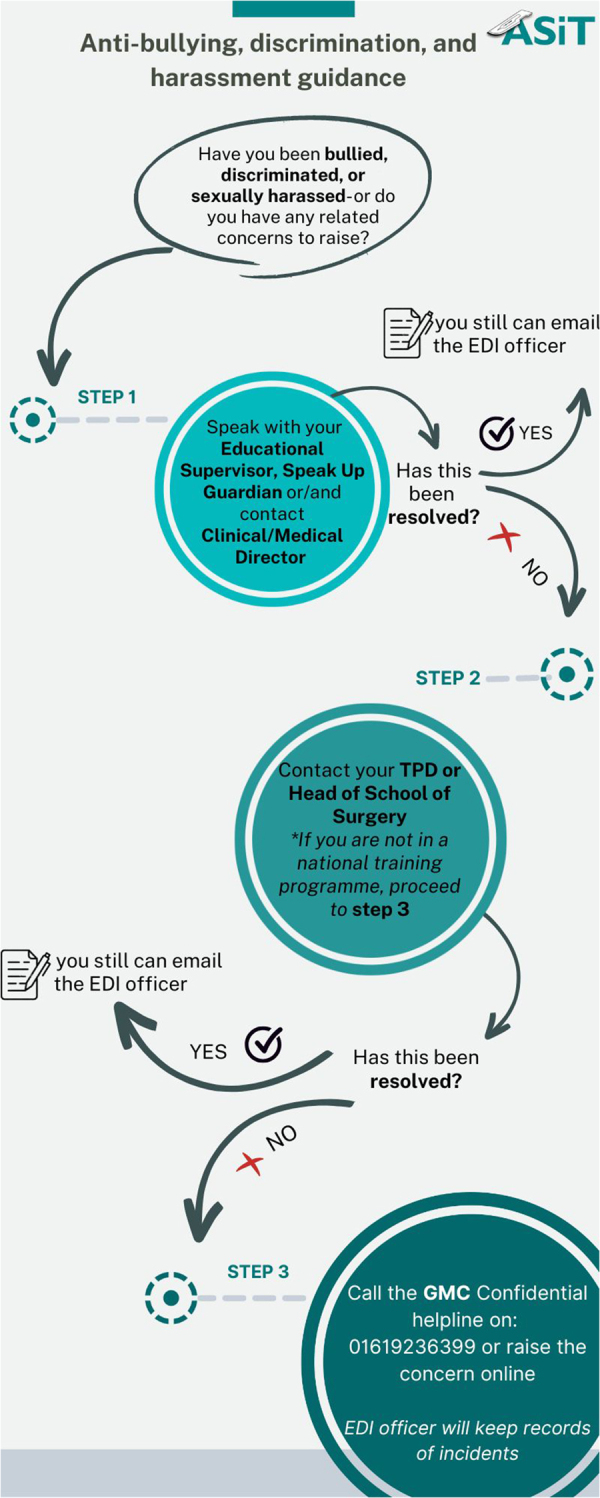
The ASiT anti-bullying, discrimination and harassment guidance

Based on our results we created an anti-BDH guidance and presented it at the breakout session at the ASiT annual conference 2023 (Figure 1).

## Discussion

To our knowledge this is the first study to identify the need for a standardised guidance, and develop an anti-BDH guidance for trainees who have experienced poor behaviours. The study was conducted among ASiT council and executive members. Our survey included different representations from all surgical subspecialties, levels of training and from different regions in the UK and Ireland. Based on the results of our survey, we created the first standardised anti-BDH guidance.

In our results, 55% of participants experienced bullying, almost 78% witnessed bullying and 67.2% did not report it. Regarding discrimination, 38% had experienced it, 62% had witnessed it and almost 69% did not report it. Sexual harassment was experienced by 24% of participants, almost 30% witnessed it and 72% did not report it. Therefore, we cannot deny the presence of poor behaviour in surgical training. However, the fact that a high percentage of trainees experienced or witnessed these misconducts and did not report them will require more attention. Previous reports mentioned that the main barriers to report were the perception that nothing would change and not wanting to be considered as a troublemaker.^3^ This was in agreement with the received comments in our survey.

It has previously been shown that there are associations between adolescent sexual assault and psychological adverse outcomes such as depression, post-traumatic stress disorder, suicide risk and poor educational outcomes.^13^ Likewise, bullied individuals are at risk for mental health problems such as psychological distress^14^ and damaged self-esteem. In addition, surgical trainees who are discriminated against, abused or harassed at least a few times per month were most likely to have symptoms of burnout and suicidal thoughts.^15^ In a previous study it was shown that surgeons are at high risk of burnout and that the suicide rate among surgeons was reported to be 13.3% or even double that of the general population, while the primary risk factor was emotional overload.^16^

Furthermore, disrespect and bullying in medicine are considered a threat to patient safety because it inhibits cooperation, which is essential to teamwork, undermines morale and cuts off all communications.^17^ Racial bias diminishes empathy, increases distrust among team members and reduces the referral rates for specialty care for persons of different colour.^9^ It is crucial to educate surgeons and staff members about the negative impact, which can affect the whole surgical-team performance. The effect of poor behaviour on the organisation impairs staff health, increases absenteeism, adds costs to the NHS, creates an upsurge in employee turnover and decreases productivity as well as requiring compensation, litigation and industrial relation costs. The consequences of bullying and harassment in the NHS was reported to cost the service in England £2.28 billion a year.^18,19^

We believe the first step of action against misconduct is to encourage victims to speak up and to seek guidance.^20^ We believe offenders need to understand the potential consequences of misconduct in terms of being suspended from their unsafe practice. These suggestions for consequences of serious misconducts and poor behaviours need confirmation from different institutions and healthcare systems.^9^ In our survey about support strategies, 74% of participants in council found lack of guidance for trainees to report poor behaviour, and 81% mentioned that they need more guidance for supporting trainees who have experienced misconduct. Almost all participants (98%) believed that surgical trainees need to know routes for reporting experiences of poor behaviours, and 88% believed that ASiT should develop a standardised pathway to support trainees against misconduct.

Our guidance started by highlighting trainees' eventual experiences of bullying, discrimination or harassment, or any related concerns to raise. We guided the trainee to report at local level, naming the clinical or educational supervisors to be approached at first, with possible escalation to the medical and clinical director. If this did not resolve the concern, we envisaged to report at regional level, including to the training director or head of the school of surgery. If this did not resolve the concern, we envisaged reporting to the General Medical Council (GMC). If the trainee is not in a structured training programme, we advised them to try to report to a local level followed by directly reporting to the GMC. There is a helpline and online reporting system, where trainees can report any poor behaviour anonymously to the GMC. We would also encourage the trainees to contact the equality, diversity and inclusion (EDI) officer to report any poor behaviour during this difficult and unpleasant experience.

The survey was anonymous, and participation was voluntary. The survey was distributed among executive members at first with a 100% response rate, followed by a one-month window for response from council members. The window took place in January, after the New Year holidays and during the annual conference preparation in March. However, we had an over 90% response rate. We believe the publication of our new anti-BDH guidance will offer structured support to trainees who have experienced poor behaviour, and it might also play an indirect role in eliminating poor behaviours in surgical training by letting the wrongdoers be aware of the possible consequences of their behaviour. We will also keep on encouraging victims of misconduct to start by speaking up to create a mentally healthier surgical training environment and a brighter future in clinical practice. As a future direction of research, a survey can be circulated among surgical trainees after the publication of the guidance to study its effect on trainees.

## Conclusions

Most of the trainees who experienced or witnessed poor behaviours did not report the incidents. Our study identified the strong need for anti-bullying, anti-discrimination and anti-harassment guidance for trainees to be able to report any poor behaviours during surgical training. The guidance was created and introduced based on our survey results. Our newly developed standardised guidance will offer trainees support for reporting misconduct, and we envisage that its use will play a role in eliminating misconducts in surgical training.
